# FOXO1 expression in keratinocytes promotes connective tissue healing

**DOI:** 10.1038/srep42834

**Published:** 2017-02-21

**Authors:** Chenying Zhang, Jason Lim, Jian Liu, Bhaskar Ponugoti, Sarah Alsadun, Chen Tian, Rameen Vafa, Dana T. Graves

**Affiliations:** 1 Department of Preventive Dentistry, Peking University School and Hospital of Stomatology, National Engineering Laboratory for Digital and Material Technology of Stomatology, Beijing Key Laboratory of Digital Stomatology, Beijing, China; 2 Department of Periodontics, School of Dental Medicine, University of Pennsylvania, Philadelphia, PA, USA; 3 The fourth affiliated hospital of Hebei Medical University, Shijiazhuang, China

## Abstract

Wound healing is complex and highly orchestrated. It is well appreciated that leukocytes, particularly macrophages, are essential for inducing the formation of new connective tissue, which requires the generation of signals that stimulate mesenchymal stem cells (MSC), myofibroblasts and fibroblasts. A key role for keratinocytes in this complex process has yet to be established. To this end, we investigated possible involvement of keratinocytes in connective tissue healing. By lineage-specific deletion of the forkhead box-O 1 (FOXO1) transcription factor, we demonstrate for the first time that keratinocytes regulate proliferation of fibroblasts and MSCs, formation of myofibroblasts and production of collagen matrix in wound healing. This stimulation is mediated by a FOXO1 induced TGFβ1/CTGF axis. The results provide direct evidence that epithelial cells play a key role in stimulating connective tissue healing through a FOXO1-dependent mechanism. Thus, FOXO1 and keratinocytes may be an important therapeutic target where healing is deficient or compromised by a fibrotic outcome.

Wound healing is complex and highly orchestrated. Normal wound healing is divided into overlapping phases of inflammation, cellular proliferation, matrix production and tissue remodeling^1^. New connective tissue provides a supporting bed for the epithelium and restores dermal function. The production of connective tissue matrix is carried out by fibroblasts and myofibroblasts that migrate from adjacent tissue or differentiate from mesenchymal stem cells (MSCs)^2^,^3^. Leukocytes, particularly macrophages are thought to be essential for inducing formation of new connective tissue^4^,^5^,^6^ by generating signals that stimulate MSCs and fibroblasts^7^. Interestingly, in some genetically modified mice and in fetal wounds there is substantially reduced numbers of macrophages without impairment of healing^8^,^9^, suggesting the existence of as yet unidentified critical mechanisms of wound healing.

Interactions between epidermal keratinocytes and dermal fibroblasts contribute to the organization of the epidermis and are thought to promote early wound healing^10^. Paracrine cross talk of cytokines between dermal fibroblasts and epidermal keratinocytes may play a role in this process^11^,^12^. During healing process re-epithelialization coincides with the recruitment of MSCs and proliferation of dermal fibroblasts^13^,^14^. The expression of growth factors, extracellular matrix constituents, proteases, and intracellular structural proteins in fibroblasts are modulated by keratinocytes when fibroblasts and keratinocytes are co-cultured^15^. In addition, myofibroblast differentiation is increased by co-culture of fibroblasts with keratinocytes^16^. This is useful to healing since myofibroblasts produce extracellular matrix and contract granulation tissue. Taken together these studies provide a framework to suggest that keratinocytes may modulate connective tissue healing but direct evidence for this concept is lacking.

We have recently shown that the forkhead box-O 1 (FOXO1) transcription factor plays a critical role in re-epithelialization *in vitro*, while FOXO3, a related FOXO transcription factor, had little to no effect^17^. FOXO1 modulates several aspects of keratinocyte behavior including resistance to oxidative stress, keratinocyte migration and is also an important regulator of broad gene expression programs, including metabolism (e.g. gluconeogenesis, amino acid catabolism, glycolysis, pentose phosphate shunt, and fatty acid/triglyceride/sterol synthesis), cell cycle, stress response, and longevity in Caenorhabditis elegans^18^,^19^. In this report, we tested the hypothesis that epithelial cell-specific FOXO1 is additionally involved in the regulation of connective tissue wound healing. Specifically, to investigate the role of cutaneous epithelium in connective tissue healing, we examined full thickness wounds in mice with keratin-14 driven FOXO1 deletion compared to littermate controls. The results demonstrate for the first time that FOXO1 activates epithelial cells to regulate proliferation of fibroblasts and MSCs, formation of myofibroblasts and production of collagen matrix. These studies provide a basis to further investigate the impact of epithelium on wound healing, which up to now has focused primarily on the effects of macrophages or the effect of connective tissue cells on epithelium. Furthermore, the results solidify the critical impact of the transcription factor FOXO1 in modulating the wound healing behavior of keratinocytes.

## Results

### Keratinocyte-specific deletion of FOXO1 impairs connective tissue healing responses in mice dermal wounds

**Figure 1:**
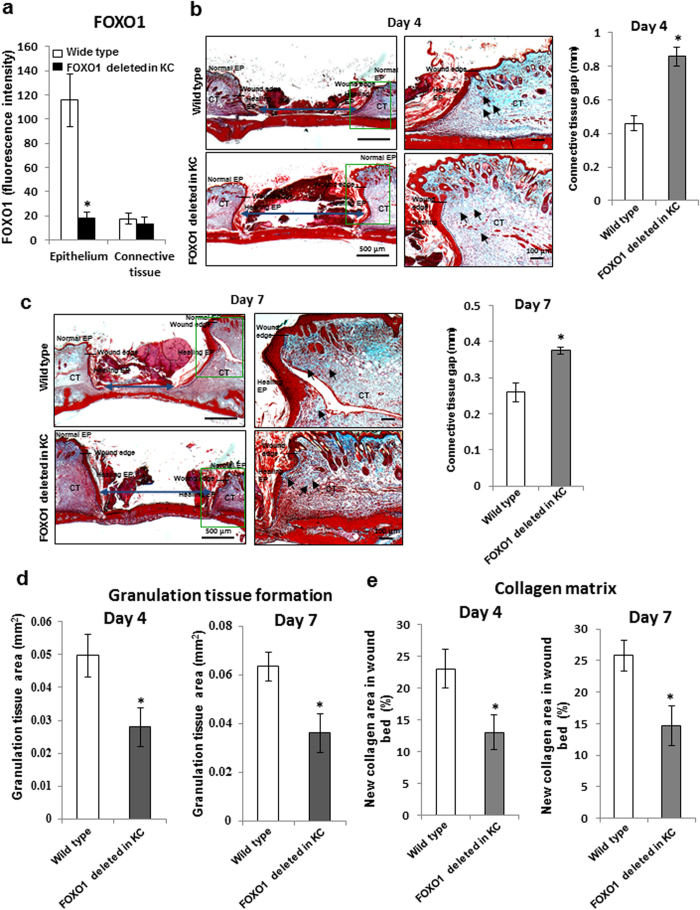
Keratinocyte-specific deletion of FOXO1 impairs connective tissue healing in dermal wounds. Dermal wounds were created in experimental mice with keratinocyte-specific FOXO1 deletion (FOXO1 deleted in KC) and littermate control (wild-type) mice. (**a**) FOXO1 expression as measured by fluorescence intensity in epithelium and connective tissue in 4 days post-wounding. (**b,c**) Representative images of Masson’s trichrome stained dermal wounds 4 and 7 days post-wounding. Bar represents 500 μm left panel and 100 μm right panel. Dotted blue lines represent healing edge of the connective tissue and the distance between them represents the connective tissue gap. Black arrows point to collagen bundles. (**d**) Area of granulation tissue formation was measured in Masson’s trichrome stained sections for day 4 and day 7 wounds. (**e**) Newly formed collagen was measured in Masson’s trichrome stained sections for day 4 and day 7 wounds. KC, keratinocytes; EP, epidermis; CT, connective tissue. n = 5–8 mice per group. *p < 0.05 vs. wild type.

To explore the role of FOXO1 in dermal wound healing, experimental mice with keratinocyte-specific FOXO1 deletion (FOXO1 deleted in KC) were compared to littermate controls (wild type). FOXO1 expression was almost 10 fold higher in the epithelium than connective tissue and reduced in epithelium by more than 85% in experimental (p < 0.05) but not control littermates (p < 0.05) (Fig. 1a). We previously demonstrated that deleting FOXO1 in keratinocytes impaired re-eppithelialization^17^. Surprisingly, the effect on connective tissue was similarly important as FOXO1 ablation in keratinocytes significantly reduced connective tissue healing (Fig. 1b,c). We examined newly formed granulation tissue and collagen matrix by Masson’s trichrome staining 4 and 7 days after wounding, time points when granulation tissue formation is robust (day 4) and a later time point when granulation tissue is present but the wound is in the process of closing (day 7) (Fig. 1b,c). At 4 days keratinocyte-specific deletion of FOXO1 reduced the amount of granulation tissue by 44% and on day 7 by 40% compared to littermate control mice (p < 0.05) (Fig. 1d). FOXO1 deletion in keratinocytes also affected the formation of collagen matrix. On day 4 the amount of collagen matrix produced was reduced by 40% with a similar reduction on day 7 in experimental mice (Fig. 1e), demonstrating the influence of keratinocytes on connective tissue wound healing.

### Keratinocyte-specific deletion of FOXO1 reduces fibroblast and MSC proliferation

**Figure 2:**
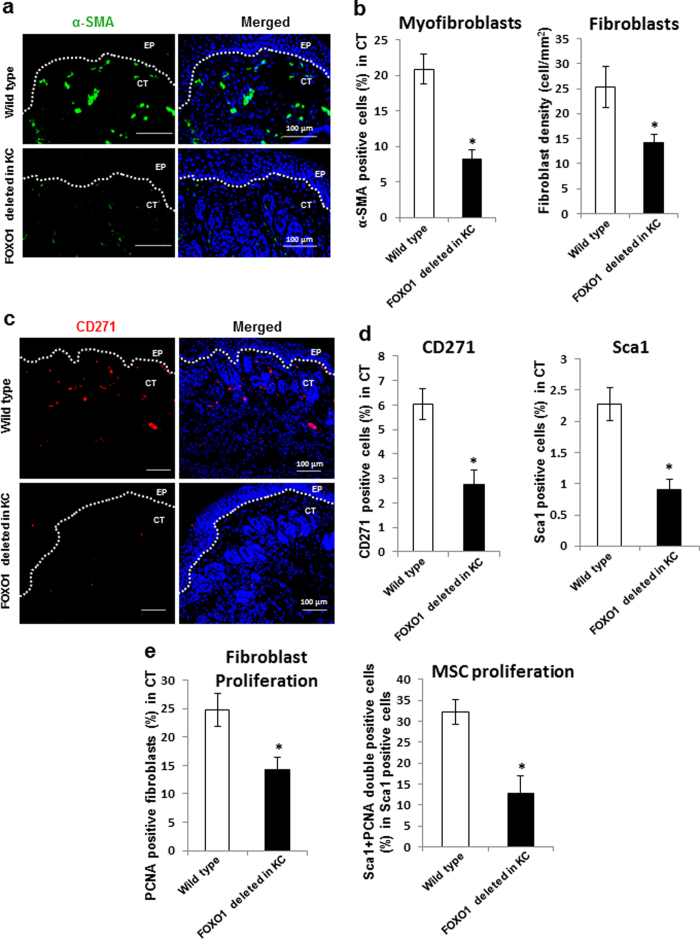
Keratinocyte-specific *FOXO1* deletion reduces fibroblast and MSC proliferation. Dermal wounds were created in experimental (FOXO1 deleted in KC) and littermate control (wild-type) mice. (**a**) Representative images of α-SMA immunofluorescence with α-SMA specific antibody on day 4 wounds. (**b**) α-SMA immunopositive cells representative of myofibroblasts in newly formed connective tissue (left panel). Fibroblasts were identified by their characteristic fusiform-shaped nucleus in hematoxylin and eosin stained sections on 4-day wounds (right panel). (**c**) CD271 immunopositive cells were identified by immunofluorescence with CD271-secific antibody 4 days after wounding. (**d**) CD271 and Sca1 immunofluorescence analyses of MSCs in the connective tissue of day 4 wounds. (**e**) PCNA immunopositive fibroblastic cells were identified by immunofluorescence with PCNA specific antibody and by characteristic appearance of fibroblast nuclei in merged images with DAPI nuclear stain (left panel). Quantification of PCNA and Sca1 double immunopositive cells for analyses of proliferating MSCs (right panel). Scale bar, 100 μm; KC, keratinocytes; EP, epidermis; CT, connective tissue. White dashed lines were the boundary between the epidermis and the dermis. n = 5–8 mice per group, *p  < 0.05 vs. wild type. Immunofluorescence was negative when a matched control antibody was used in lieu of specific antibody.

The effect of epithelial-specific FOXO1 deletion on myofibroblast was then assessed *in vivo* by immunofluorescent staining for α-smooth muscle actin (α-SMA)^20^ (Fig. 2a). Myofibroblasts play a crucial role in wound contraction during healing. The number of myofibroblasts was reduced 60% by FOXO1 deletion in epithelium (p < 0.05) (Fig. 2b). The impact of epithelial-specific FOXO1 deletion on fibroblast numbers was then assessed *in vivo* in histologic sections. Day 4 was chosen since it reflects a time point of robust connective tissue formation. Fibroblast density in connective tissue was reduced by 44% (p < 0.05) (Fig. 2b).

The impact of keratinocyte-specific FOXO1 deletion on cells that express MSC markers in connective tissue was then measured by assessing the number of CD271 and stem cell antigen1 (Sca1) immunopositive cells^21^. CD271 positive cells were reduced by 55% in keratinocyte-FOXO1 null mice compared to control littermates (p < 0.05) (Fig. 2c,d). Sca1 positive cells were reduced by 60% in keratinocyte-FOXO1 null mice (p < 0.05) (Fig. 2d). The number of proliferating fibroblasts was measured by assessment of proliferating cell nuclear antigen (PCNA) and were reduced by 42% in keratinocyte-FOXO1 null mice compared to littermate control mice (p < 0.05) (Fig. 2e), indicating impaired fibroblast proliferation with epithelial-specific FOXO1 deletion. The number of proliferating Sca-1 immunopositive cells was reduced by 60% in the connective of keratinocyte-FOXO1 null mice compared to littermate control (p < 0.05) (Fig. 2e), indicating that FOXO1 expression in keratinocytes promotes MSCs proliferation.

### The effect of keratinocyte-specific *FOXO1* deletion on connective tissue formation *in vivo* is mediated by TGFβ1

**Figure 3:**
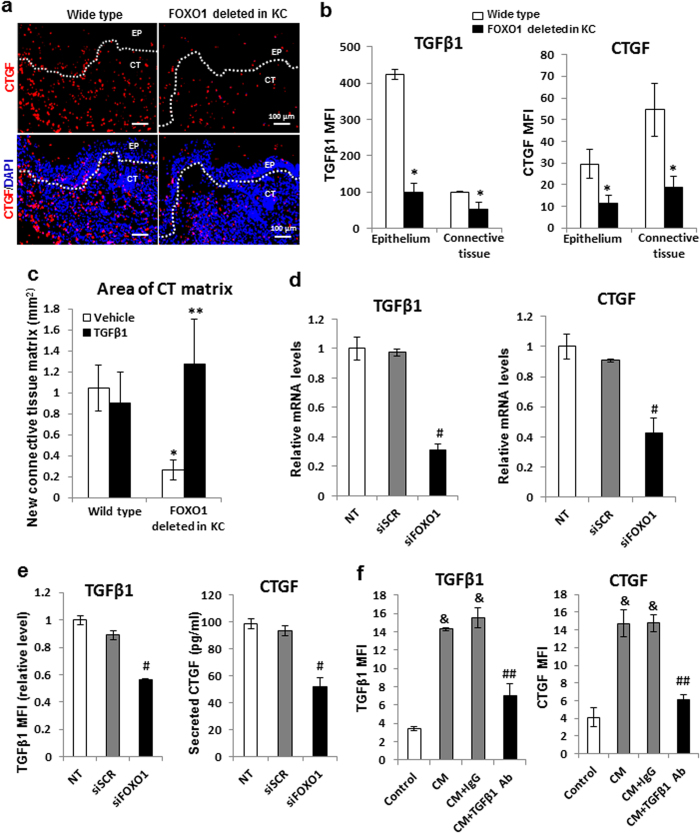
FOXO1 activity in keratinocytes drives TGFβ1 expression in healing epithelium and connective tissue growth factor expression in healing connective tissue. (**a**) CTGF expression was identified with specific CTGF antibody by immunofluorescence in 4-day wounds. Scale bar, 100 μm. (**b**) TGFβ1 and CTGF expression was identified with a specific antibody to each by immunofluorescence in day 4 wounds. Expression was determined by mean immunofluorescence intensity (MFI). (**c**) Quantification of new connective tissue formation by Masson’s trichrome stain of day 4 wounds with or without TGFβ1 treatment. (**d**) qRT-PCR analysis of TGFβ1 and CTGF expression in primary cultures of normal human epidermal keratinocytes (NHEK). (**e**) TGFβ1 expression was assessed in primary fibroblast cultures with specific antibody by measuring mean immunofluorescence intensity and CTGF by ELISA. Fibroblasts were incubated with keratinocyte-conditioned medium transfected in NHEK cells that had been transfected with FOXO1 siRNA (siFOXO1) or scrambled siRNA (siSCR). (**f**) TGFβ1 and CTGF immunofluorescence analyses of fibroblasts treated with keratinocyte-conditioned medium with TGFβ1 blocking antibody or matched control IgG. For all immunofluorescence experiments, the images were negative when a matched control antibody was used in lieu of specific antibody. MFI, mean fluorescence intensity; KC, keratinocytes; EP, epidermis; CT, connective tissues. White dashed lines were the boundary between the epidermis and the dermis. n = 5–8 mice per group. *p < 0.05 vs. matched wild type, **p < 0.05 vs. matched vehicle group, ^#^*p* < 0.05 vs. scrambled siRNA, ^&^*p* < 0.05 vs. control group, ^##^*p* < 0.05 vs. matched IgG control.

Connective tissue growth factor (CTGF also known as CCN2) plays an important role in fibroblast behavior during wound healing^22^ and is induced by transforming growth factor β1 (TGFβ1)^17^. Due to the importance of TGFβ1^23^ and CTGF^22^ in healing we determined whether *FOXO1* deletion in epithelial cells affected TGFβ1 and CTGF expression in the epithelium and connective tissue by standardized quantitative immunofluorescence measuring mean fluorescence intensity in each compartment (Fig. 3a). TGFβ1 expression was four-fold higher in the wounded epithelium than connective tissue (Fig. 3b). As expected, expression of TGFβ1 in epithelium was FOXO1 dependent based on our previous results. Surprisingly, TGFβ1 expression in the connective tissue compartment was reduced by ~60% when *FOXO1* was absent in epithelia (Fig. 3b), indicating that keratinocytes influence TGFβ1 expression in connective tissue.

In contrast to TGFβ1, CTGF expression in connective tissue following wounding was 2 fold higher than epithelium (Fig. 3b). CTGF levels in the epithelium of keratinocyte-FOXO1 null mice were reduced by 61% in experimental mice. CTGF levels in connective tissue were also dependent upon cutaneous epithelium and were reduced by 66% when *FOXO1* was ablated in these cells (Fig. 3b). Thus, keratinocytes have a significant role in regulating the expression of CTGF in connective tissue. The linkage between keratinocyte-specific *FOXO1* deletion, TGFβ1 and connective tissue was reinforced by rescue of the deficit in connective tissue formation in experimental mice but not control mice treated with exogenous TGFβ1 (Fig. 3c). Thus, deficiency caused by keratinocyte-specific *FOXO1* deletion is overcome by addition of TGFβ1 to wounds while this addition has little effect in normal littermate controls, indicating that keratinocyte-produced TGFβ1 is needed for optimal connective tissue healing.

The mechanisms through which keratinocytes may regulate fibroblasts and MSCs were examined *in vitro*. FOXO1 silencing in normal human epidermal keratinocytes (NHEK) *in vitro* significantly reduced TGFβ1 and CTGF mRNA levels (Fig. 3d), consistent with the data obtained from *in vivo* (Fig. 3b). Moreover, FOXO1 knockdown in keratinocytes reduced the capacity of epithelial cells to stimulate TGFβ1 expression in fibroblasts by almost 40% and CTGF production by 45% (p < 0.05) (Fig. 3e). Keratinocyte stimulation of TGFβ1 and CTGF expression in fibroblasts was blocked by antibody to TGFβ1 (*p* < 0.05) (Fig. 3f). Thus, keratinocytes stimulate CTGF expression via TGFβ1 that is mediated by FOXO1, consistent with reports that fibroblasts are an important source of CTGF in dermal wound healing^22^.

### Inhibition of TGFβ1 and CTGF blocks fibroblast and MSC proliferation, myofibroblast differentiation, and collagen production *in vitro*

**Figure 4:**
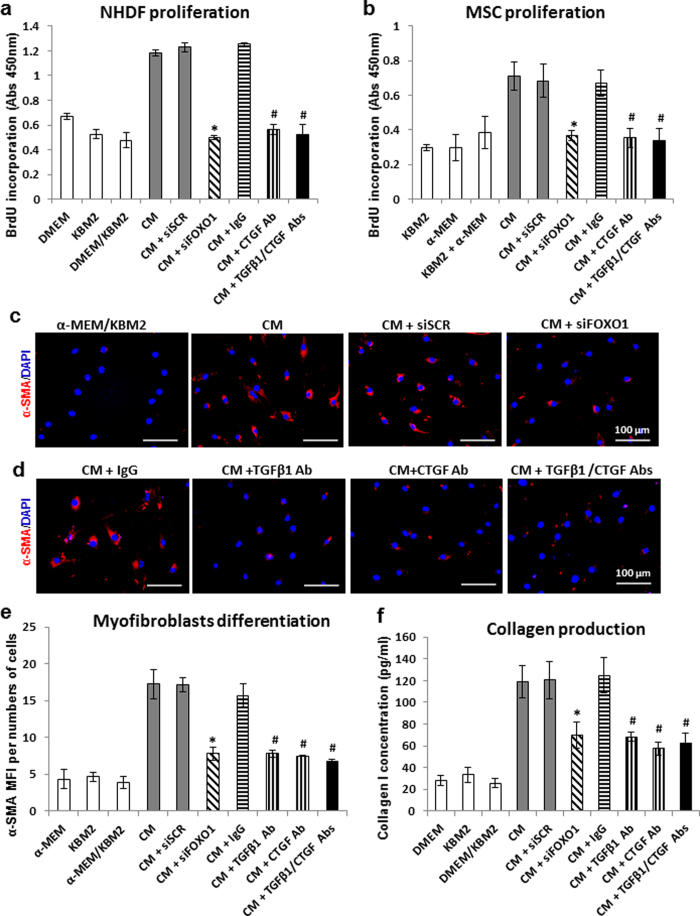
Keratinocyte stimulation of fibroblasts and MSCs is dependent on TGFβ1 and CTGF. Primary dermal fibroblasts (**a**,**f**) and MSCs (**b**–**e**) were incubated with conditioned medium collected from keratinocytes transfected with FOXO1 or scrambled siRNA, then incubated with TGFβ1 and/or CTGF blocking antibody or matched control IgG. (**a**,**b**) DNA synthesis of dermal fibroblasts (**a**) and MSCs (**b**) were determined by BrdU incorporation. (**c**) Representative images of α-SMA immunofluorescent staining for MSCs after treated with conditioned medium collected from keratinocytes transfected with FOXO1 or scrambled siRNA. (**d**) Representative images of α-SMA immunofluorescent staining for MSCs after treated with keratinocyte conditioned medium and TGFβ1 and/or CTGF blocking antibody. Scale bar, 100 μm. (**e**) The quantification of α-SMA protein expression by immunofluorescence in MSCs. Data is expressed as mean fluorescence intensity (MFI) per number of cells. (**f**) Collagen I levels in NHDF cells was measured by means of ELISA. *p < 0.05 compared to siSCR, ^**#**^*p* < 0.05 vs. matched IgG group.

The role of keratinocytes in regulating fibroblasts and MSCs through FOXO1 was explored further. Keratinocytes stimulated fibroblast and MSC proliferation as determined by BrdU incorporation and this increase was blocked by FOXO1 knockdown (Fig. 4a,b). The increase stimulated by keratinocytes was inhibited by antibody to CTGF (Fig. 4a,b). The combination of TGFβ1 and CTGF blocking antibodies displayed a similar effect as CTGF antibody alone. Since TGFβ1 stimulates fibroblasts in part through CTGF^24^, the results indicate that CTGF is a critical factor by which keratinocytes regulate fibroblasts through a mechanism mediated by FOXO1.

The effect of FOXO1 in modulating keratinocyte regulated MSC differentiation was also investigated. Keratinocytes induced a 4-fold increase in MSC differentiation to myofibroblasts determined by α-SMA expression and 70% of the induction effect was blocked by FOXO1 knockdown (Fig. 4c,e). Moreover, formation of myofibroblasts was dependent upon the TGFβ1/CTGF axis as it was blocked by antibody specific for CTGF or TGFβ1 (p < 0.05) (Fig. 4d,e). Cutaneous epithelial cells also stimulated a 4-fold increase in collagen I production examined by enzyme-linked immunosorbent assay (ELISA), which was reduced in half by FOXO1 knockdown compared to scrambled siRNA (p < 0.05) and dependent upon TGFβ1 and CTGF (p < 0.05) (Fig. 4f). These studies provide further evidence that FOXO1 regulated TGFβ1 and CTGF production represents an important mechanism through which epithelia modulate connective tissue healing.

## Discussion

The cellular and molecular mechanisms that underlie tissue repair are incompletely understood and represent a significant health care issue^1^. Studies presented here establish for the first time *in vivo* that cutaneous epithelia play a key role in the repair of wounded connective tissue and that the transcription factor FOXO1 is essential for up-regulating this effect. When *FOXO1* is specifically deleted in cutaneous epithelial cells there is a ~40–50% reduction in granulation tissue formation and production of extracellular matrix. This is equivalent to the substantial reduction reported for macrophage deletion and indicates the importance of activating cutaneous epithelial cells to promote connective tissue healing^4^,^5^,^6^.

It has been postulated that interactions between epidermal keratinocytes and dermal fibroblasts contribute to wound healing. The predominant line of thinking has been that cells and growth factors in connective tissue modulate proliferation and migration of keratinocytes to promote re-epithelialization^25^,^26^. This is supported by evidence that specific deletion of Rac1 in fibroblasts found in connective tissue *in vivo* negatively impacts re-epithelialization^27^. In contrast, less is known about the control of connective tissue by epithelial cells although *in vitro* studies suggest that factors produced by these cells may affect fibroblast activity^15^,^16^. Thus, our *in vivo* results provide the first direct evidence that keratinocytes modulate the repair of connective tissue and support previous *in vitro* studies that paracrine factors released by keratinocytes contribute to connective tissue healing^28^,^29^.

Mesenchymal cells such as fibroblasts are essential to dermal wound healing. In addition to producing connective tissue, fibroblasts provide signals to enhance repair^30^. The healing of a wound requires the generation of well-orchestrated signals that stimulate MSCs and fibroblasts^31^. Within the wound bed, fibroblasts produce collagen and other extracellular matrix constituents. We found that *FOXO1* deletion in epithelium led to impaired healing that included decreased formation of new connective tissue. Fibroblast numbers and proliferation were both modulated by keratinocytes in a FOXO1 dependent mechanism, consistent with impaired production of extracellular matrix. In addition keratinocyte-specific *FOXO1* deletion led to reduced MSCs numbers and proliferation, which may contribute to reduced myofibroblast numbers seen in our study, and have secondary effects on subsequent healing events^32^.

TGFβ1 is an important regulator of connective tissue healing stimulating the production of ECM proteins such as collagen and initiating granulation tissue formation^33^. TGFβ stimulates the expression of CTGF, which is thought to be responsible for many of the pro-fibrotic properties of TGFβ, particularly in the promotion of fibroblast proliferation and ECM production^24^,^34^. CTGF plays a physiological role in early wound healing^35^. Many different cell types including fibroblasts, epithelial cells, endothelial cells, and vascular smooth muscle cells express CTGF^36^. In wound repair *in vivo* there is a coordinated up-regulation of TGFβ1 followed by CTGF expression^22^. We found that TGFβ1 and CTGF expression in both epithelium and connective tissue were decreased by keratinocyte-specific deletion of *FOXO1. In vitro* knockdown of FOXO1 in keratinocytes also reduced TGFβ1 and CTGF expression in keratinocytes. Antibody blocking studies demonstrated that TGFβ1 produced by keratinocytes up-regulates CTGF in fibroblasts, and *FOXO1*-deletion in keratinocytes results in deficient connective tissue formation. In addition, we found that keratinocyte could induce MSCs to differentiate into myofibroblasts *in vitro*, which was blocked by inhibition of TGFβ1 and/or CTGF. This is consistent with the other report that CTGF could direct fibroblast differentiation from human mesenchymal stem/stromal cells *in vitro*^2^,^34^. It is also possible that keratinocyte produced CTGF contributes to connective tissue healing.

**Figure 5:**
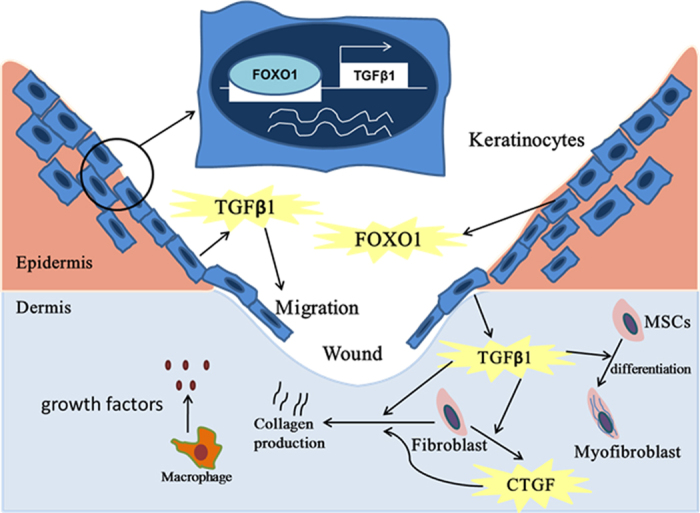
FOXO1 organizes keratinocyte activity to promote connective tissue healing. During wound healing FOXO1 binds to the TGFβ1 promoter in keratinocytes to up-regulate TGFβ1 expression, which promotes connective tissue wound healing directly and also induces CTGF production. TGFβ1 and CTGF stimulate differentiation of myofibroblasts and connective tissue formation. In addition, TGFβ1 stimulates keratinocyte migration to enhance re-epithelialization. Leukocytes, particularly macrophages also produce growth factors to enhance healing.

In summary, we found that keratinocytes played a significant role in regulating connective tissue healing through a mechanism that was dependent upon FOXO1. Moreover, a key component of this mechanism involved TGFβ1, which is produced in sub-optimal amounts when *FOXO1* is deleted in keratinocytes as shown by improved healing when TGFβ1 is applied to experimental mice with keratinocyte-specific *FOXO1* deletion but not in normal control littermates. Combined with our previous results^17^, we propose that FOXO1 activation induces release of TGFβ1 by keratinocytes to stimulate re-epithelialization^17^ and that TGFβ1 up-regulates CTGF expression to promote connective tissue healing by stimulating fibroblasts, myofibroblasts and cells that express mesenchymal stem cell markers (see Fig. 5). Our finding indicates that keratinocytes are also important as a cellular source of factors that drive connective tissue repair similar to macrophage-derived growth factors that stimulate fibroblast proliferation, myofibroblast differentiation and extracellular matrix synthesis^4^.

## Material and Methods

### Animal model

Experiments were approved by the University of Pennsylvania Institutional Animal Care and Use Committee and all methods were performed in accordance with guidelines and regulations relevant to the study. Mice with floxed *FOXO1* were generously provided by Ronald A. DePinho (MD Anderson Cancer Center, Houston, TX) as previously described^37^. Mice expressing Cre recombinase [strain Tg (KRT14-cre)1Amc/J] were obtained from the Jackson Laboratory (Bar Harbor, ME). Keratinocyte-specific *FOXO1* deletion was obtained by crossing the above two mentioned mice to generate experimental (FOXO1 deleted in KC) and control littermates. All the experiments were performed with adult mice age of 16–20 weeks.

### Skin wounding experiment

Mice were anesthetized by i.p. administration of ketamine (80 mg/kg) and xylazine (5 mg/kg). The scalp was shaved and cleansed with isopropyl alcohol and prepared for surgery. Two excisional wounds were made with a 2mm sterile biopsy punch in the scalp to create a circular full-thickness wounds as described previously^38^. Wounds were harvested on days 4 and 7 post-wounding. Wound healing process was assessed by quantification of the calibrated digital photographs. The wound areas were calculated from the photographs using Nikon NIS-elements D image analysis software (Nikon). For TGFβ1 treatment experiment, 100ng of recombinant TGFβ1 (PeproTech) in 20 μl of 3% methylcellulose vehicle or vehicle alone was topically applied to the wounds every other day from the time of wounding. In most *in vivo* experiments, 6–8 mice were examined per group.

### H&E staining and Masson’s trichrome staining

The scalp and attached calvarial bone were fixed in 4% paraformaldehyde overnight and decalcified in 10% EDTA solution. Five-micrometer sections were stained using hematoxylin & eosin (H&E) for microscopic evaluation of the epithelial gap and the granulation tissue gap using Nikon NIS-elements image analysis software (Nikon) at the center of each lesion. The connective tissue gap was measured by drawing parallel lines along the edge of the connective tissue adjacent to the open wound and the distance between them was measured. Granulation tissue formation was measured in Masson’s trichrome stained sections as highly cellular tissue which stained red. Recently formed collagen matrix stains light blue compared to dark blue mature collagen matrix. The wound edges were determined by use of histologic landmarks including the position of hair follicles and thickened epithelium and the floor of the wound was bordered by the osseous floor or muscle bundles. Newly formed collagen matrix in the wound area was measured with Nikon NIS-elements image analysis software. Collagen matrix was identified by its characteristic color in Masson’s trichrome stained sections.

### Immunofluorescent staining *in vivo*

Immunofluorescent staining was performed on paraffin sections that were fixed with 4% paraformaldehyde sectioned at 4 microns. After blocking with nonspecific binding blocking buffer (Millipore), tissue sections were incubated with the appropriate primary antibodies followed by biotinylated secondary antibody (Vector Laboratories). Sections were subsequently incubated with avidin-biotin-peroxidase enzyme complex (Vector Laboratories) and followed by tyramide signal amplification (PerkinElmer) to enhance the fluorescent signal. Nuclei were stained with DAPI. Primary antibodies used were Sca1 (Abcam), CD271 (Abcam), CTGF (Santa Cruz Biotechnology Inc.), TGFβ1 (Abcam) and α-SMA (Abcam). Images were captured using a fluorescence microscope (ECLIPSE 90i, Nikon). Images for control antibodies gave negative immunostaining. Nikon NIS Elements AR image analysis software (Nikon) was used to do image analysis. Percent positive cells were calculated from the number of immunopositive cells divided by the number of DAPI positive cells. To examine expression levels mean fluorescence intensity was measured. In each case, a standardized region of interest was defined in each specimen and was of similar size. After the region of interest was selected the exposure time was set to obtain results in the linear range.

### Cell culture

Primary normal human epidermal keratinocytes (NHEK) were purchased from Lonza and cultured in KGM-2 growth medium in the presence of human keratinocyte growth supplements (Lonza) and antibiotics (Life Technologies). For experiments, insulin was excluded from the culture media to prevent inactivation of FOXO1. NHDFs were maintained in DMEM medium containing 10% fetal bovine serum (FBS) (Gibco, life technologies), while hBMSCs were maintained in α-MEM medium supplemented with 10% FBS. All the cell cultures were maintained in 5% CO_2_/air and used between passages P1–P4.

### Conditioned Media Experiments

The effect of keratinocyte-conditioned media (CM) on TGFβ1 and CTGF expression in normal human dermal fibroblasts (NHDF) was performed as follows. In cases where CM was collected from transfected cells, conditioned media was collected from transfected keratinocytes 24 hours after transfection with FOXO1 siRNA or scrambled control siRNA (Dharmacon) using GenMute siRNA Transfection Reagent (SignaGen Labs). Cells were incubated with 5–10 nM siRNA with transfection reagent for six hours and transferred to standard medium for 16 hours. Cells were transferred to fresh media for the collection of conditioned media. To assess the impact of conditioned media on fibroblasts or human bone marrow-derived mesenchymal stem cells (hBMSCs) the conditioned media was diluted 1:1 with α-MEM medium supplemented with 1% FBS. Therefore, the fibroblasts were incubated in low-serum (total 0.5% FBS) when stimulated with keratinocyte conditioned media. For antibody neutralization studies, anti-TGFβ1^39^ (10 μg/ml, R&D Systems) or anti-CTGF^40^ blocking antibody (10 μg/ml, Santa Cruz) was added 1 hour prior to incubation of keratinocyte-conditioned media with NHDF or hBMSCs for 3 days.

### Immunofluorescent staining *in vitro*

Cells were fixed briefly with 10% formalin, permeabilized with 0.5% Triton X-100 and incubated overnight at 4 °C with primary antibodies to TGFβ1 (Abcam), CTGF (Santa Cruz), α-SMA (Abcam) or an equal amount of matched negative control IgG, then with the corresponding secondary antibody. Nuclei were stained with DAPI. Images were captured with a fluorescence microscope (Nikon) and the mean fluorescence intensity (MFI) was determined using NIS-Elements AR software (Nikon).

### BrdU incorporation Assay

The effect of keratinocyte-CM on the proliferation of fibroblasts and MSCs were determined with a BrdU Cell Proliferation Assay Kit (Cell Signaling Technology). CM were then collected from keratinocytes transfected with FOXO1 siRNA or scrambled control siRNA, and incubated with fibroblasts or MSCs in 96-well plates with/without TGFβ1 antibody (R&D Systems) or/and CTGF blocking antibody (Santa Cruz) for 72 hours, and incubated with 10 μM BrdU for 6 hours. Cells were fixed with cold methanol and incubated with anti-BrdU antibody. BrdU incorporation was determined by measuring absorbance at 450 nm. Experiments were repeated at least three times.

### Real-time PCR

Total RNA was isolated from NHEK cells transfected with FOXO1 or scrambled siRNA using an RNeasy kit (Qiagen). After reverse transcription (Applied Biosystems), real-time quantitative PCR (qPCR) for was performed with Taqman system (Roche Diagnostics). Results were normalized with values obtained from ribosomal protein RPL32, a ribosomal protein. Experiments were repeated three to four times.

### ELISA

CTGF levels in media from fibroblasts treated with keratinocyte-CM were determined by measuring the levels of CTGF in media using specific ELISA kit (PeproTech). Collagen levels in fibroblasts were measured as follows. Fibroblasts were first starved in 0.1% FBS for 24 hours, then treated with keratinocyte-CM with/without TGFβ1 (R&D Systems) or/and CTGF blocking antibody (Santa Cruz) for 72 hours. Supernatant was collected to measure collagen by ELISA (Biomatik).

### Statistics

All data are reported as the mean ± standard deviation. Group mean values were compared, as appropriate, by Student’s two-tailed t-test or one-way ANOVA with Scheffe’s post-hoc test. A p-value < 0.05 was considered significant.

## Additional Information

**How to cite this article**: Zhang, C. *et al*. FOXO1 expression in keratinocytes promotes connective tissue healing. *Sci. Rep.* **7**, 42834; doi: 10.1038/srep42834 (2017).

**Publisher's note:** Springer Nature remains neutral with regard to jurisdictional claims in published maps and institutional affiliations.
